# Impact of duration of silicone oil tamponade on foveal and parafoveal thickness in rhegmatogenous retinal detachment: a retrospective cohort study

**DOI:** 10.1007/s10792-024-03096-8

**Published:** 2024-04-02

**Authors:** Wael Ahmed Ewais, Lamia Samy Ali, Fayrouz M. Aboalazayem

**Affiliations:** Kasr Alainy Faculty of Medicine, Cairo University, 108 Alshaheed Ahmed Hamdy St., Madkour, Giza, Cairo Egypt

**Keywords:** Silicone oil tamponade, Central foveal thickness, Parafoveal thickness, OCT

## Abstract

**Purpose:**

To detect and analyze the influence of the duration of tamponade with silicone oil on the foveal and parafoveal thickness in cases of rhegmatogenous retinal detachment.

**Methods:**

This is a retrospective cohort study of 64 eyes with rhegmatogenous retinal detachment in one eye who underwent pars plana vitrectomy (PPV) with silicone oil injection during the period between January 2019 and December 2021. The patients were divided into 2 groups. Group A with early removal of the silicone oil after 3–4 months and Group B with late removal of the silicone oil (SOR) after 6–8 months. The 2 groups were compared as regards the central foveal (CFT) and parafoveal (PFT) thickness changes from baseline, just before SOR, and after SOR. Changes in best corrected visual acuity (BCVA), relative risk of severe thinning. It was conducted at Kasr Alainy Hospital.

**Results:**

64 eyes were enrolled in the study. Group A included 36 eyes, and group B included 28 eyes. The mean CFT changed insignificantly from 253 ± 52 μm to 252 ± 48 μm after SOR in group A; while it changed significantly from 211 ± 52 μm to 202 ± 46 μm after SOR in group B. The mean PFT decreased insignificantly from 299 ± 39 μm to 297 ± 40 μm in group A, while it decreased significantly from 284 ± 46 μm to 273 ± 44 μm in group B. Lines of improvement of BCVA were 4.11 ± 1.88 in group A, and 2.00 ± 1.24 in group B. Relative risk of severe foveal thinning after SOR was 14.3, and severe parafoveal thinning was 15.43, in group B compared to group A.

**Conclusion:**

Longer period of silicone oil tamponade may carry a higher risk for severe foveal and parafoveal thinning after silicone oil removal.

**Trial registration:**

The study was registered at clinical trial.gov under the title of (Duration of silicone oil tamponade on foveal and parafoveal thickness in Rhegmatogenous Retinal Detachment) with an ID NCT05817630 at April 2023 “retrospectively registered”.

## Introduction

Silicone oil (SO) is one of the major tamponading agents, that are used during pars plana vitrectomy for eyes with rhegmatogenous retinal detachment (RRD). It is used with high efficacy in eyes with either simple or complex RRD [1–6].

Silicone oil (SO) is a viscid water repellant oil, which is chemically inert. It is composed of polydimethylsiloxane (PDMS), with a range of viscosity between 1000 and 30,000 cst, most commonly; 1000, 2000, and 5000 cst. It has a low specific gravity, low buoyancy, and low interfacial tension; that pushes it high above water [7, 8].

Silicone oil is considered a permanent tamponading agent, as it is not spontaneously absorbed. On the contrary; it should be surgically removed. Duration of tamponade is commonly around 3 months, but can range from a few weeks to 12 months [1, 4, 9].

Macular thinning under silicone oil has been reported; where there had been gradual thinning until just before silicone oil removal (SOR). This had been followed by some restoration of macular thickness over a varied period of time after SOR [10–15].

More detailed observations had been performed on macular thickness changes; like segmental analysis of the macular layers [16, 17], and description of foveal and parafoveal changes in thickness [18]. However; the impact of duration of silicone oil tamponade on macular thickness wasn’t clearly highlighted [16–18].

Our study observes and analyses the influence of the duration of tamponade with silicone oil on the foveal and parafoveal thickness.

## Patients/materials and methods

### Study design

This is a retrospective cohort study which received approval from the ethics committee of Kasr Alainy medical school, and was registered at clinical trial.gov under the title of (Duration of silicone oil tamponade on foveal and parafoveal thickness in Rhegmatogenous Retinal Detachment) with an ID NCT05817630. All procedures fulfilled declaration of Helsinki. Written informed consent was obtained from each subject at the time of the intervention. The study was conducted at kasr Alainy hospital.

We reviewed the records of patients who underwent a 23-gauge PPV for RRD and were left with a SO endotamponade during the period from January 2019 to December 2021 at Kasr Alainy Hospital of Cairo University.

**Inclusion Criteria** were cases of fresh macula-OFF retinal detachment with: (1) age > 18 years old; (2) Silicone oil tamponade (SO) 2000 centistokes (cst) for 3–8 months (3) Normal fellow eye (4) attached retina under SO and for 3 months after SOR (5) normal intraocular pressure (IOP < 22 mmHg; with or without anti-glaucoma treatment) all over the period from baseline till 3 months after SOR.

**Exclusion Criteria** were (1) Rhegmatogenous Retinal Detachment (RRD) with other tamponades like Gas or Air or heavy silicone oil (2) SO tamponade less than 3 months, or more than 8 months; (4) Recurrent retinal detachment (RD) under SO and up to 3 months after SOR; (5) Vitrectomized fellow eye and (6) Retinal detachment, or macular disease in the fellow eye (7) macular pathology under SO or after SOR ( Epimacular membranes, macular hole, cystoid macular edema), (8) myopia > 6.0 diopters (9) axial length > 26 mm and (10) refractive difference between both eyes more than 1 diopter (11) Glaucoma and ocular hypertension (IOP > 22 mmHg with or without anti-glaucoma treatment) at any time from vitrectomy till 3 months after SOR, (12) giant retinal tear, (13) proliferative vitreoretinopathy (PVR), and (14) macula-ON RD, (15) cases where silicone oil 1000 or 5000 cst had been used.

The patients were divided into 2 groups based on the duration of SO tamponade:

- **Group A**: (Short duration) patient who underwent early SOR around 3 months post operatively (3-4 months).
- **Group B**: (Long duration) patients who underwent late SOR at around 6 months post operatively (6-8 months)

### Optical coherence tomography (OCT)

All OCT images (using OCT Spectralis, Heidelberg, Germany), were carefully reviewed to exclude patients with insufficient data and poor-quality images. In addition, any preexisting or newly developed macular pathologies were observed. Therefore, we also excluded patients with ERM, CME, macular hole, and subretinal fluid in the treated and/or fellow eye. Image analysis was performed in the operated eye and the fellow healthy eye in each patient over the study period. The fellow eye was used as the baseline measurement of CFT and PFT; instead of the operated eye because of the difficulty of obtaining a proper image for a detached retina, and the possibility of the presence of macular edema in the detached retina. Fellow eye was chosen for baseline measurements after checking the exclusion criteria based on the assumption that there had been no refractive difference and no macular pathologies respectively. This was accomplished by: checking the glasses prescriptions, checking the axial length before PPV and during history taking from the patients. Central foveal thickness (CFT) and para foveal thickness (PFT) were measured in the fellow eye at baseline, then in the operated eye just before SOR and 3 months after SOR. PFT was measured as the average value of measurements of the 4 sectors in the inner circle around the fovea.

### Outcome measures

Primary outcome measures were the change in CFT and PFT between baseline (fellow eye), under SO just before SOR, and 3 months after SOR for both groups A and B. Secondary outcome measures were both: Changes in logMAR best corrected visual acuity (BCVA) between baseline (operated eye, not the fellow eye), and 3 months after SOR for both groups A and B. Relative risk (RR) of severe foveal and parafoveal thinning after SOR in both groups.

We defined severe foveal thinning as a change in the CFT between the baseline fellow eye and the operated eye after SOR ≥ 3* μ*m. We defined severe parafoveal thinning as change in the PFT between the baseline fellow eye and the operated eye after SOR ≥ 5* μ*m. These definitions were based on the average measurements that have been demonstrated in previous studies (10–15).

### Statistical analysis

All statistical analyses were performed using StatPlus software (StatPlus: mac LE, build 6.1.2/Core v6.1.0, AnalystSoft Inc., Walnut, CA, USA). The baseline characteristics, CFT, and PFT records were analyzed using descriptive analyses. Comparison between continuous variables; in each group; at baseline, before SOR, and after SOR; was done using paired sample t-test. ANOVA Test was used to compare all 3 data records (baseline, before SOR, and after SOR). ANOVA test was used to compare the parafoveal subsectors just before SOR as well. Chi Square test was used to compare between categorical variables (e.g., number of eyes with mild versus severe foveal and parafoveal thinning after SOR respectively in both groups). Linear regression analysis, was used to correlate changes in BCVA with changes in CFT and PFT between both groups respectively. Relative risk was calculated for severe CFT and PFT thinning using the relative risk formula. Statistical significance was set at P value less than 0.05.

## Results

We enrolled 64 eyes of 64 patients who underwent PPV for RRD with SO tamponade after checking the inclusion and exclusion criteria respectively. The mean age was 42 ± 12 years. They included 30 females and 34 males, (*p* = 0.754). All the eyes had macula-OFF RD. They were divided into 2 groups, Group A included eyes which underwent early SOR after 3–4 months and Group B included eyes that underwent late SOR after 6–8 months. The baseline demographic data and clinical characteristics are summarized in Table 1. All eyes had been injected with 2000 cst Silicone oil at the end of the PPV for RRD. All surgeries had been performed by a single surgeon (Wael A. Ewais).

**Table 1:** Baseline demographic and clinical data

Data	Group A	Group B	*p* Value
Number of eyes	36	28	
Gender			
Male	20	14	0.754
Female	16	14	
Age (years)	42 ± 14	40 ± 11.2	0.701
Duration of symptoms (days)	6.32 ± 1.1	6.47 ± 0.9	0.98
Refractive status (diopters)	−2.25 ± 0.5	−4.75 ± 0.75	0.08
log MAR BCVA	1.39 ± 0.55	1.63 ± 0.32	0.13
IOP (mmHg)	12.44 ± 2.01	13.00 ± 1.88	0.42
CFT of fellow eye (u)	238 ± 74	211 ± 52	0.23
PFT of fellow eye (u)	299 ± 39	284 ± 84	0.329

*BCVA* Best corrected visual acuity; *Log MAR* Logarithm minimal angle of resolution; *IOP* Intraocular pressure; *CFT* central foveal thickness; *PFT* Para foveal thickness; *u* microns

### The central foveal thickness

In group (A): The mean CFT decreased significantly from a baseline 253 ± 52 μm (fellow eye) to 240 ± 45 *μ*m under SO just before SOR (*p* = 0.03), then it increased significantly back to 252 ± 48 μm 3 months after SOR (*p* = 0.02). However; the change from baseline till after SOR was statistically insignificant (*p* = 0.48) (Table 2).

**Table 2:** Central foveal thickness changes in both groups

CFT (u)	Group A	*p* Value	Group B	*p* Value
initial (fellow)	253		211	
Before SOR	211	0.03	195	0.0008
Before SOR	211		195	
After SOR	252	0.02	202	0.006
initial (fellow)	253		211	
After SOR	252	0.48	202	0.01

*CFT* central foveal thickness; *SOR* Silicone oil removal

In group (B): The mean CFT decreased significantly from a baseline 211 ± 52 μm (fellow eye) to 195 ± 59 *μ*m under SO just before SOR (*p* = 0.0004), then it increased significantly back to 202 ± 46 μm 3 months after SOR (*p* = 0.01). The change from baseline till after SOR was statistically significant as well (*p* = 0.01) (Table 2).

**Table 3:** Parafoveal thickness changes in both groups

PFT (u)	Group A	*p* Value	Group B	*p* Value
Initial (fellow)	299		284	
Before SOR	277	< 0.0001	254	< 0.0001
Before SOR	277		254	
After SOR	297	< 0.0001	272	0.0003
initial (fellow)	299		284	
After SOR	297	0.26	272	0.0001

*PFT* Parafoveal thickness; *SOR* silicone oil removal

There is a statistically significant greater foveal thinning (from baseline to 3 months after SOR) in group B (mean 8.64 μm) than in group A (mean 1.0 μm) (*p* = 0.03). However, we cannot confirm that there is a statistically significant difference in foveal thinning between baseline and under SO in both groups (*p* = 0.99), and in foveal thickening after SOR between both groups (*p* = 0.49).

### The parafoveal thickness

In group (A): The mean PFT decreased significantly from a baseline 299 ± 39 μm (fellow eye) to an average 277 ± 33 μm under SO just before SOR, then it increased back to 297 ± 40 μm after SOR (*p* = 0.0001). However; the change from baseline till after SOR was statistically insignificant (*p* = 0.26). Before SOR; there was a statistically insignificant difference between PFT in all the 4 subsectors; superior was 276 ± 28 u, inferior was 282 ± 31 u, temporal was 267 ± 34 u, and nasal was 272 ± 33 u (*p* = 0.79) Table 3.

In group (B): The mean PFT decreased significantly from a baseline 284 ± 46 *μ*m (fellow eye) to an average 254 ± 51 μm under SO just before SOR, then it increased back to 273 ± 44 *μ*m after SOR (*p* = 0.0001). The change from baseline till after SOR was statistically significant as well (*p* = 0.0001). Before SOR; there was a statistically insignificant difference between PFT in all the 4 subsectors; superior was 252 ± 48 u, inferior was 261 ± 52 u, temporal was 246 ± 51 u, and nasal was 251 ± 48 u (*p* = 0.83). Table 3.

There is a statistically significant greater parafoveal thinning (from baseline to 3 months after SOR) in group B (mean 10.86 μm) than in group A (mean 1.89 μm) (*p* = 0.02). However; we cannot confirm that there is a statistically significant difference in parafoveal thinning between baseline and under SO in both groups (*p* = 0.12), and in parafoveal thickening after SOR between both groups (*p* = 0.80).

### Central foveal thinning versus parafoveal thinning

There was a statistically insignificant difference between central foveal thinning and parafoveal thinning in both groups A and B respectively (Group A: *p* = 0.59) (Group B: *p* = 0.56).

### Best corrected visual acuity (BCVA)

In group (A): BCVA improved significantly from a baseline logMAR 1.39 ± 0.55 to log MAR 0.47 ± 0.25 at 3 months after SOR (*p* < 0.0001).

In group (B): BCVA improved significantly from a baseline logMAR 1.63 ± 0.32 to log MAR 1.01 ± 0.33 at 3 months after SOR (*p* < 0.0001).

Group A eyes had a statistically significant better logMAR BCVA at 3 months after SOR than group B eyes (*p* < 0.0001). There is a statistically significant more lines of improvement of BCVA in group A (4.11 ± 1.88 lines) than in group B (2.00 ± 1.24 lines) (*p* = 0.0006).

### Changes in BCVA versus changes in CFT and PFT

We confirm that there was a statistically significant strong correlation between changes in BCVA and changes in CFT and PFT in both groups A and B respectively (Table 4).

**Table 4:** Correlation between visual change and changes in CFT and PFT respectively

	Group A		Group B	
	R value	*p* Value	R value	*p* Value
BCVA—CFT changes	0.04	< 0.0001	0.23	0.0002
BCVA—PFT changes	0.119	< 0.0001	0.144	0.001

*BCVA* Best corrected visual acuity; *CFT* central foveal thickness; *PFT* parafoveal thickness

### Relative risk of severe central foveal thinning:

There was a statistically significant higher relative risk (14.1) of severe foveal thinning after SOR in group B than in group A (*p* = 0.0001).

We found that in group A; 5.56% of the eyes had severe foveal thinning after SOR. While in group B; 78.57% of the eyes had severe foveal thinning after SOR. Table 5

### Relative risk of severe para foveal thinning:

There was a statistically significant higher relative risk (15.43) of severe parafoveal thinning after SOR in group B than in group A (*p* = 0.0001). Table 5

**Table 5:** Relative Risk (RR) of severe macular thinning

Severe Thinning (from baseline till after SOR)	u	Relative Risk (group B compared to Group A)	*p* Value
CFT	> 3	14.1	0.001
PFT	> 5	15.3	< 0.0001

*SOR* Silicone oil removal; *CFT* Central foveal thickness; *PFT* Parafoveal thickness; *RR* Relative Risk

We found that in group A 5.56% of the eyes had severe parafoveal thinning after SOR. While in group B; 85.71% of the eyes had severe parafoveal thinning after SOR.

### Intraocular pressure

There was a statistically insignificant difference in intraocular pressure (IOP); at both baseline and at 3 months after SOR; between both groups (*p* = 0.31). In each group there was a statistically insignificant change in IOP between baseline and at 3 months after SOR (group A: *p* = 0.27) (group B: *p* = 0.16). We did not observe optic disc pallor; either before or after SOR; in any of our cases.

## Discussion

Our study compares changes in foveal and parafoveal thickness between short duration of silicone oil (SO) tamponade around 3 months and long duration of SO tamponade around 6 months, compares visual outcomes in both time frames, and calculates the relative risk of severe foveal and parafoveal thinning with both time frames.

Previous studies didn’t focus on duration of SO tamponade as a predictor factor for postoperative changes in macular thickness. These studies described the gradual macular thinning till just before SOR (silicone oil removal), which was followed by partial restoration of macular thickness over a period of time after SOR [10–14]. Lee JW etal mentioned data about foveal and parafoveal changes under SO, but didn’t compare the differences in foveal and parafoveal thickness between different durations of SO tamponade [18].

Lee JW etal reported a change in PFT from a mean 307 μm to a mean 295 μm after SOR (SO tamponade < 3 months), and from a mean 319 μm to a mean 303 μm after SOR (SO tamponade > 3 months) [18]. However, they didn’t compare the PFT changes between both time frames (subgroups). Moreover, they mentioned data about the parafovea only (not about the fovea) [18].

On the other hand, we compared both CFT and PFT changes between both the early and late SOR groups.

*Duration of SO tamponade* in our retrospective study comprises a range of 3–8 months; broadly divided into a short duration of 3–4 months, and a long duration of 6–8 months. This can be attributed to the retrospective nature of the study, and the protocol that is applied for management of cases of RD that are operated in our hospital. The general rule in our hospital is SO tamponade using 2000 cst silicone oil for around 3 months. However; a delay in SOR may happen because of non-compliance with follow-up by the patients, or a long waiting list for SOR. Intended delay of SOR; for 6 months or more; may be done for cases with PVR or giant retinal tears; which were excluded from our study to avoid bias and confounding factors.

*We defined severe foveal and parafoveal thinning* in our study based on the data about average CFT and PFT that had been previously reported in previous studies [10–15, 18]. Lee JW etal performed a retrospective study in which they published data about average thickness of both the fovea and the parafovea at baseline and after SOR for a sample of 40 eyes. They reported a reduction in CFT from a baseline 251.8 μm to 235 μm just before SOR then to 251.5 μm 3 months after SOR, and a reduction in PFT from a baseline 311 μm to 279 μm just before SOR then to 306 μm 3 months after SOR [18]. We considered the average changes in CFT and PFT as a border line value between mild and severe changes in CFT and PFT. That was the background for considering ≥ 3 μm change in CFT as a severe foveal thinning after SOR, and ≥ 5 μm change in PFT as a severe parafoveal thinning after SOR.

Foveal thinning can be explained by the macula-OFF retinal detachment itself, in addition to mechanical compression by the globular configuration of the silicone oil (SO) bubble. Macula-Off retinal detachment is a direct precursor for macular atrophy. All eyes in our study were macula-OFF RD to eliminate macula status as a confounding variable between both groups. Macula-off RD in all the cases in our study; makes mechanical compression the main cause of foveal thinning with both the short and long durations of SO tamponade respectively. Hostovsky A and coauthors 2021 observed several OCT patterns for macula-Off RD; including: intraretinal splitting with cystoid cavities, intraretinal splitting with undulated outer retina, subretinal deposits, and variable foveal detachment heights that would all be biomarkers for visual prognosis after surgery [19].

Parafoveal thinning (PFT) maybe related to the SO tamponade itself. Parafoveal area is anatomically convex; and it comes in touch with the convex SO globule (especially in prone position). This may elicit a permanent mechanical compression and a probable disturbance of macular blood flow [7, 8, 10–14, 18].

The inferior subsectors of PFT were not thinner than the superior subsectors of PFT in our study. This is due to the fact that silicone oil bubble is large enough (with complete fill) to be in contact with the whole macular area, the superior retina, and part of the inferior retina below the inferior arcades.

*Long duration of SO tamponade* is associated with 14.1 relative risk of severe persistent Foveal thinning and 15.43 relative risk of Parafoveal thinning in our study. We assume that a long duration around 6–8 months may provide an additional explanation for the probable mechanisms of visual loss under SO. Time under SO enhances accumulation of electrolytes like potassium which is toxic to the retinal nerve fiber layer. Long duration of SO tamponade fills the vitreous cavity for a long time; providing little space for fluids; thus, eliciting a prolonged dehydration effect on the macula. Long duration of SO tamponade may aggravate both the mechanical compression effect of the SO globule for both the fovea and parafovea, and the vascular insufficiency for the parafoveal area [7, 8, 10–14, 18]. However; we did not observe more thinning in nasal subsectors than temporal subsectors of parafoveal areas in our study; which is usually related to ganglion cell affection by both mechanical and vascular perfusion factors.

Visual rehabilitation in our study is worse with long duration of SO tamponade than with short duration of SO tamponade. Moreover; there is a relatively strong correlation between visual rehabilitation and reduction of CFT and PFT in both time frames. This may refer to a probable impact for foveal and parafoveal thinning on visual function.

Intraocular pressure (IOP) was controlled whether with or without treatment in all the enrolled eyes in our study. All enrolled eyes were a macula-OFF RD; and none of the cases had a complex RD (e.g., giant retinal tear, or PVR). All the surgeries had been performed by a single surgeon (Wael A. Ewais), and SO 2000 cst had been used in all cases; which helped eliminating confounding variables from influencing our results for both short and long durations of SO tamponade.

The fellow eye was used as the baseline measurement of CFT and PFT; instead of the operated eye because of the difficulty of obtaining a proper OCT image for a detached retina, and the possibility of the presence of macular edema in the detached retina. That’s why all the enrolled eyes were macula-OFF RD. Macula-ON RD was chosen as one of the exclusion criteria in our study to standardize the baseline measurement using the fellow eye.

We had a baseline CFT difference between both groups (average 253 u in group A, and 211 u in group B), which was statistically insignificant. This can be attributed to presence of more myopic eyes in group B than in group A (−2.25 ± 0.5 D in group B, −4.75 ± 0.75 D in group B, *p* value 0.08). Despite similar duration of symptoms in both groups, we cannot rely completely on these data since it is subjectively reported by our patients.

Limitations of our study include the retrospective nature, and the relatively small sample size. We identified a range of time for both short and long SO tamponades; not a specific prospectively identified time for both groups; to overcome the limitation of the retrospective design. The small sample size is due to the strict exclusion criteria in our study that eliminates possible confounding factors for the study.

Further studies may be needed to assess the impact of duration of SO on macular thickness that would be prospective randomized clinical trials, that would be performed for longer durations of follow up, on complex RD cases (PVR and giant retinal tears), and on a larger sample size. Further studies using OCTA would be needed to assess vascular perfusion factors for macular thinning under SO.

## Conclusion

Long duration of SO tamponade may carry a higher risk of severe foveal and parafoveal thinning than short duration of SO tamponade; and it may be associated with a worse visual outcome. We wouldn’t recommend a long duration of SO tamponade for Retinal detachment.
